# COVID Mortality Prediction with Machine Learning Methods: A Systematic Review and Critical Appraisal

**DOI:** 10.3390/jpm11090893

**Published:** 2021-09-07

**Authors:** Francesca Bottino, Emanuela Tagliente, Luca Pasquini, Alberto Di Napoli, Martina Lucignani, Lorenzo Figà-Talamanca, Antonio Napolitano

**Affiliations:** 1 Medical Physics Department Bambino Gesù Children’s Hospital, Scientific Institute for Research, Hospitalization and Healthcare (IRCCS), 00165 Rome, Italy; martina.lucignani@opbg.net; 2 Neuroradiology Unit, NESMOS Department, Sant’Andrea Hospital, La Sapienza University, 00165 Rome, Italy; lucapasquini3@gmail.com (L.P.); adnapoli7@hotmail.com (A.D.N.); 3 Neuroradiology Service, Radiology Department, Memorial Sloan Kettering Cancer Center, New York, NY 1275, USA; 4 Radiology Department, Castelli Romani Hospital, 00040 Ariccia (RM), Italy; 5 Neuroradiology Unit, Imaging Department, Bambino Gesù Children’s Hospital, Scientific Institute for Research, Hospitalization and Healthcare (IRCCS), 00165 Rome, Italy; lorenzo.figatalamanca@opbg.net

**Keywords:** machine learning, deep learning, COVID, mortality, prediction, imaging, computer Tomography (CT)

## Abstract

More than a year has passed since the report of the first case of coronavirus disease 2019 (COVID), and increasing deaths continue to occur. Minimizing the time required for resource allocation and clinical decision making, such as triage, choice of ventilation modes and admission to the intensive care unit is important. Machine learning techniques are acquiring an increasingly sought-after role in predicting the outcome of COVID patients. Particularly, the use of baseline machine learning techniques is rapidly developing in COVID mortality prediction, since a mortality prediction model could rapidly and effectively help clinical decision-making for COVID patients at imminent risk of death. Recent studies reviewed predictive models for SARS-CoV-2 diagnosis, severity, length of hospital stay, intensive care unit admission or mechanical ventilation modes outcomes; however, systematic reviews focused on prediction of COVID mortality outcome with machine learning methods are lacking in the literature. The present review looked into the studies that implemented machine learning, including deep learning, methods in COVID mortality prediction thus trying to present the existing published literature and to provide possible explanations of the best results that the studies obtained. The study also discussed challenging aspects of current studies, providing suggestions for future developments.

## 1. Introduction

More than a year has passed since the report of the first case of coronavirus disease 2019 (COVID), and many deaths continue to occur. Despite the discovery of different vaccine formulas from different pharmaceutical companies, many problems related to mass production and distribution across the world still persist. This factor is accompanied by political and economic constraints that may further limit vaccine access [1]. For these reasons, pandemic containment is a hard task, resulting in increased deaths. At the time this manuscript is written, SARS-CoV-2 numbers reported by the World Health Organization (Ginevra, Switzerland) (https://covid19.who.int/, 31 May 2021) worldwide include: almost 173,005,553 people infected with SARS-CoV-2; more than 3,727,605 death cases and around 1,900,955,505 vaccine doses administered. Multiple hospitalizations, due to the rapid spread of the virus have required an improvement of patient management throughout the healthcare system. In this context, it is important to minimize the time required for resource allocation and clinical decision making, such as triage, choice of ventilation modality, admission to the intensive care unit. Currently, baseline machine learning (ML) and deep learning (DL) techniques are widely accepted thanks to their ability to obtain information from the input data without “a priori” definitions [2]. These approaches can be efficiently tested in healthcare applications such as diagnosis of diseases, analysis of medical images, collection of big data, research and clinical trials, management of smart health records, prediction of outbreaks [3]. Consequently, DL models are capable of solving complex tasks in the intricate clinical field [4]. ML is acquiring an increasingly sought-after role in predicting the outcome of COVID patients [3,5,6,7]. For instance, a mortality prediction model could rapidly and effectively help clinical decision-making for COVID patients at imminent risk of death. Recent studies reviewed predictive models for SARS-CoV-2 diagnosis and severity, length of hospital stay, intensive care unit (ICU) admission, mechanical ventilation modality outcomes [8,9,10,11,12], highlighting pitfalls of the machine and deep learning methods based on imaging data [13]; however, systematic reviews focused on prediction of COVID mortality outcome with ML methods, including DL techniques, are lacking in the literature.

The aim of this review is to discuss the current state of the art of ML methods to predict COVID mortality by: (1) summarizing the existing published literature on baseline ML- and DL-based COVID mortality prognosis systems based on medical evaluations, laboratory exams and Computer Tomography (CT); (2) presenting relevant information including the type of data employed, the data splitting technique, the proposed ML methodology and evaluation metrics; (3) providing possible explanations of the best results obtained; (4) discussing challenging aspects of current studies, providing suggestions for future developments.

## 2. Literature Review Methods

This systematic review considers the state of the art in ML and DL as applied to COVID mortality prediction. We performed a MEDLINE search on PubMed on 26 May 2021 using the terms “machine learning covid survival” (146 results), “machine learning covid mortality” (131 results), “deep learning covid survival” (49 results), “deep learning covid mortality” (45 results) and additional similar terms. The search results were filtered to remove: duplicates, ML approaches for SARS-CoV-2 diagnosis or prognosis besides mortality, preprint works, abstract works, papers that deviated from our purpose. We try to shed some light on peculiar characteristics of these studies in terms of: (i) data source, (ii) data partitioning, (iii) class of features, (iv) implemented features ranking method, (v) implemented ML technique, (vi) metrics evaluated for performance assessment.

### 2.1. Data Source

We emphasized the study location and whether the dataset of each study was public or private, single site or multicenter.

### 2.2. Data Partitioning

We focused on the type of model validation that each study used to split data into train and test groups. Particularly, we chose to report the number of subjects used for the train and test set, and the corresponding number of survived and non-survived subjects. Additionally, we categorized validations type in: internal, external, merged and prospective (in particular internal prospective or merged prospective); referring to Internal validation when the studies subdivided a single-site database into train and test groups; external validation when studies trained and tested the model using data from independent cohorts, obtained from different sites. Moreover, we referred to merged validation for studies that combined data from different sites producing a single database to split into train and test groups or used multisite publicly available epidemiological datasets. Finally, we indicated prospective validation when studies implemented a temporal validation, assessing temporal generalizability. In the case of internal prospective validation, data of hospitalized patients from a first timeframe was used for training and data of patients admitted at a different time from the same hospital was used for testing. Differently, prospective merged validation relied on multisite data to train the model and multisite data collected in a subsequent timeframe for testing.

### 2.3. Class of Features

We expected to collect papers with both clinical and imaging features. In the latter, we included hand-made extracted features with radiomic analysis and the features learned with the use of convolutional neural networks (CNN). Clinical features comprise demographic (e.g., age, sex, race), comorbidities (e.g., diabetes, heart disease), symptoms (e.g., cough, fever), vital signs (e.g., heart rate, oxygen saturation), laboratory values (e.g., glucose, creatinine, haemoglobin), disease treatment and clinical course (e.g., artificial ventilation, length of hospital stay, drugs). Clinical features can be classified in binary (yes/no: 0/1) and continuous features (numerical values). We considered binary features when studies associated them with 0/1 values or dichotomized continuous feature’s value in a binary form, defining a numerical range and setting the feature to 1 if the value is within that range, 0 otherwise. While we have referred to continuous features when studies used predictors (features used for prediction tasks) as continuous variables or dichotomized binary features in continuous features.

### 2.4. Implemented Features Ranking Method

To build a reliable model for solving classification, the feature set should contain as much useful information as possible, and a number of features as small as possible. It is necessary to filter out the irrelevant and redundant features by choosing a subset of relevant features to avoid over-fitting and tackle the problem of dimensionality [14]. Feature ranking (or selection or reduction) techniques are a good approach for features space dimensionality reduction [15]. Feature ranking improves features understanding and reduces the computational cost, increasing the efficiency of the classification. Since Shapley Additive Explanation (SHAP) and least absolute shrinkage and selection operator (LASSO) logistic regression algorithm are widely used methods for model interpretation and feature selection in survival studies [16,17,18,19], we highlighted whether the studies used these methods or others. Particularly SHAP is a method to explain individual predictions by computing the contribution of each feature to the prediction. LASSO is a new method for estimation in linear models based on regression analysis.

### 2.5. Implemented ML Techniques

With the aim of identifying the most used and performing methods, we focused on the prediction technique used in each work, highlighting whether it belonged to baseline ML or advanced DL algorithms. Since in literature there are many implementable and customizable algorithms, we expected to find several and different methods employed in the works included in this review. However, we expected to find techniques attributable to one of the following four classes, according to the characterized basic algorithm: (i) regression, (ii) classifier, (iii) neural network and (iv) ensemble learners. Particularly, we included in regression the algorithms that estimate the model function from the input features to numerical or continuous output. In classifiers, we included the algorithms that estimate the model function from the input features to discrete or categorical output. In neural networks, we comprehend architectures inspired by the neurons in the brain. Finally, we consider the ensemble models that combine several base models. In addition to the algorithm, we aimed to identify the K-fold cross-validation used in each work, a statistical method used to estimate the skill of a model, with k referring to the time of validations execution to reinforce the validity of the model.

### 2.6. Metrics

We highlighted the measures that each selected study reported to evaluate model performance, including Accuracy (ACC), Area Under the Curve—Receiving operator characteristic (AUC-ROC), Area Under the Precision-Recall Curve (AU-PRC); Sensitivity (SENS), Specificity (SPEC), Positive Predictive Value (PPV), Negative Predictive Value (NPV), F1-score, Matthew Correlation Coefficient (MCC), Balanced Accuracy (B-ACC).

ACC is the fraction of predictions the model performed right:

$$\;\mathrm{ACC}=\frac{\mathrm{TP}+\mathrm{TN}}{\mathrm{TP}+\mathrm{TN}+\mathrm{FP}+\mathrm{FN}}$$

AUC-ROC provides an aggregate measure of performance across all possible classification thresholds;

AU-PRC can be used to test all the possible positive predictive values and sensitivities obtained through a binary classification;

SENS is the ability of a model to detect a true positive:

$$\mathrm{SENS}=\frac{\mathrm{TP}}{\mathrm{TP}+\mathrm{FP}};$$

SPEC is the ability of a model to detect a true negative:

$$\mathrm{SPEC}=\frac{\mathrm{TN}}{\mathrm{TN}+\mathrm{FP}};$$

PPV is the model ability in not categorizing some people as having the condition when in fact they do not:

$$\mathrm{PPV}=\frac{\mathrm{TP}}{\mathrm{TP}+\mathrm{FP}};$$

NPV is the ability of a model in not categorizing some people as not having the condition when in fact they do:

$$\mathrm{NPV}=\frac{\mathrm{TN}}{\mathrm{TN}+\mathrm{FN}};$$

F1—score is defined as the harmonic mean of precision and recall:

$$F_{1}-\mathrm{score}=\frac{2\times \mathrm{TP}}{2\times \mathrm{TP}+\mathrm{FP}+\mathrm{FN}};$$

MCC is a measure not influenced by the unbalanced nature of a dataset:

$$\mathrm{MCC}=\frac{\mathrm{TP}\times \mathrm{TN}-\mathrm{FP}\times \mathrm{FN}}{\sqrt{\left(\mathrm{TP}+\mathrm{FP}\right)\times \left(\mathrm{TP}+\mathrm{FN}\right)\times \left(\mathrm{TN}+\mathrm{FP}\right)\times \left(\mathrm{TN}+\mathrm{FN}\right)}};$$

B-ACC is a metric that evaluates binary classifier performance considering the imbalanced dataset:

$$B-\mathrm{ACC}=\frac{\mathrm{SENS}+\mathrm{SPEC}}{2}.$$

where TP, FP, TN, FN are respectively true positive, false positive, true negative and false negative.

## 3. Literature Review Results

Twenty-four papers were included for discussion in this work. Out of these, 3 were DL papers, 17 traditional ML papers and 4 hybrid papers.

### 3.1. Data Source

Public datasets were used by 2/24 papers [20,21] (Supplementary Table S1). Private data were used in 22/24 papers, with 9/24 using data from a single site and 13/24 using multicentric data. A total of 22/24 studies used data from a single country: 8/24 from China, 8/24 from the United States, 2/24 from the United Kingdom, 2/24 from Korea, 2/24 from Italy, 2/24 studies used data from more than one country: including Italy, Spain and the United States [22], and Iran and the United States [23] (Table 1).

### 3.2. Data Partitioning

In Table 1 we show the type of model validation that each study used to split data into train and test groups, indicating the number of subjects and the corresponding number of survived and non-survived subjects. Internal validation was performed in 15/24 studies [24,25,26,27,29,30,31,32,33,35,36,37,38,39,40,42].

A total of 7/24 studies performed external validation [22,28,41,43].

A total of 7/24 studies performed a merged validation, particularly 4 of these combined data from different sites producing a single database [22,28,41,43] and 3 of these used multisite publicly available epidemiological datasets [20,21,44].

A total of 2/24 studies implemented internal prospective validation [24,38] and 3/24 studies implemented a prospective merged validation [24,40,43].

### 3.3. Class of Features

A total of 2/24 studies used CT imaging features (Ning et al., 2020; X. Fang et al., 2021).Particularly, Ning et al. used CT images in addition to clinical features, while Fang et al. developed an artificial intelligence (AI) framework using deep neural networks to segment lung lobes and pulmonary opacities, and baseline ML methods to predict mortality based on radiological severity scores (accounting for the volume ratio of pulmonary opacities in each lung lobe).

A total of 19/24 studies adopted binary features [20,21,22,24,25,26,27,30,31,32,33,34,35,36,37,38,40,41,42,43]. 1/24 study dichotomized continuous feature’s value in a binary form [28].

A total of 16/24 studies adopted continuous features [21,22,24,27,29,30,31,32,33,35,37,38,40,41,42,43]. A total of 2/24 studies dichotomized binary feature in continuous feature associating a Charlson comorbidity score to the feature’s value [39,40].

In Table 2 we show features type and class, feature ranking techniques, features dimension reduction included in each study and the most important features derived.

### 3.4. Implemented Features Ranking Method

Most studies used a high number of starting features [24,27,29,30,31,32,33,35,37,38,39].

We found 8/24 articles in which SHAP method was used to optimize survival prediction in COVID [22,24,25,26,29,32,39,40,41,44]. Vaid et al. demonstrated that interactions between features had a weak contribution to outcome prediction compared to the importance of each feature individually [24]. On the contrary, Abdullal et al. used SHAP analysis to assess the contribution of patient variables to the mortality prediction, with no features reduction [25,26]. A similar approach was employed by other studies [22,29,32]. Subudhi et al. tested 18 models and performed the SHAP technique on the temporally distinct patients to compare the important features selected on the different validation cohorts [40]. In the other works, the most relevant features were selected with LASSO [24,33,34,35]. Ko et al. employed the analysis of variance (ANOVA) to select features with the most significant difference between survivors and deceased. Particularly, in the study by Ko et al., the purpose was to identify a significant difference between the two classes (survived and no survived) by selecting the features with *p*-values less than 10^−5^ [27]. In the study by Di et al., the moDel Agnostic Language for Exploration and eXplanation (DALEX) package is used as a features selection method usually adopted for predictive models. Booth et al. implemented a different ranking method including a Logistic Regressor (LR) classifier, obtaining regression coefficients as a measure of feature importance]. An et al. compared different features ranking models to figure out if there was a coherence in using different features ranking procedures [34]. Hu et al. used regression algorithms for feature reduction as well [37]. Li et al. used the univariate analysis to compare distribution differences between COVID survivors and non-survivors [30]. Moreover, they compared an evaluation model with 83 features and a model with only the first five features selected. Yan et al. performed feature ranking with a Multi-tree XGBoost [38]. With DL models, features selection can be implemented by combining available features, as shown by Zhu et al. [31], to obtain the optimal number of features necessary for classification. Three articles did not apply any feature selection before the prediction algorithm [20,21,36].

### 3.5. Implemented ML Techniques

Based on our classification of ML methods above mentioned, we reported in this section the particular algorithm implemented in each study and we exploited the most relevant characteristics:

#### 3.5.1. Regression Methods

A total of 8/24 papers evaluated LR performance and compared them with the performance of other ML tools tools [20,22,23,24,30,35,37,43].According to Li et al., and compared to other methods, LR models are superior in terms of high-speed calculation and easy-to-interpret results, which might enhance their clinical applications [30]. Furthermore, Li et al. developed a novel LR modeling method that ensured the training of optimal predictors only (the adopted method for feature selection will be explored in-depth in the next paragraph) [30]. LR is one of the traditional regression techniques, widely used to observe the risk conditions among exposure and disease [45].

Abdulaal et al. implemented a Cox regression model, an algorithm used for multivariable analysis, starting from predictors chosen in concordance with previous literature. In the training phase, the variables not significantly associated with mortality were eliminated [26]. The model assessment was performed with the final Cox model, re-trained on the entire training set. A total of 2/24 studies [24,34] implemented LASSO [19]. An et al. tested several machine-learning algorithms, including LASSO [34]. Likewise, Vaid et al. employed LASSO for data training [24]. A total of 1/24 studies proposed a partial least square (PLS) regression model [37], which is a very versatile, supervised method purposely used to address the issue of making good predictions in multivariate problems [34]. PLS is based on a mixture of linear regression models [46], to reduce the complexity of high-dimension class prediction problems.

#### 3.5.2. Classifiers

A total of 6/24 papers [20,23,29,34,42,43] used a Support vector machine (SVM) for mortality prediction, a nonlinear statistical data supervised ML tool that achieved high performance in survival prediction tasks [3,47]. Subudhi et al. used Support Vector Classifier (SVC), a linear supervised ML algorithm [40].

The studies by An et al. and Subudhi et al. also included k-nearest neighbors (KNN) [34,40], considered the oldest and simplest method for pattern classification [48]. Moreover, An et al. reported the features associated with mortality as input data on multivariate Cox regression [34].

Furthermore, Yun Li et al. employed outliers detection algorithms to isolate samples that deviate in the dataset [20].

Subudhi et al. evaluated 18 baseline ML algorithms, including linear models such as: Passive Aggressive Classifier, a Stochastic gradient descent classifier, and a perceptron classifier. The Authors also included Gaussian Naïve Bayes, a supervised algorithm based on Bayes theorem and Decision Tree classifier, a non-parametric supervised algorithm. Moreover, Subudhi et al. used models based on discriminant analysis (DA) such as linear DA and quadratic DA with linear and quadratic decision boundary, respectively, which are specific techniques that classify based on similarities between elements [40].

#### 3.5.3. Neural Networks

5/24 papers developed neural network architectures [21,25,31,36]. Abdullal et al. used a feature set as input for the ANN architecture: the input layer dimension was equal to the number of patient features (*n* = 22) [25]. Information was then fed to two densely connected hidden layers, consisting of one-dimensional vectors placed in cascade. Each layer had the aim of creating increasingly meaningful representations of the input data before attempting outcome prediction. Zhu et al. implemented a deep neural network of six fully connected dense layers, whose input layer had 53 features, for predicting survival [31] In this work, features ranking was made with the permutation importance methodology, training 6-layer DNNs with 5-fold cross-validation. Once the top five clinical variables were selected, the neural network was reduced to a simple 2-layer DNNs, to prevent overfitting.

1/24 paper [21] tested convolutional neural networks for unsupervised features extraction from CT images, to predict patient mortality. Ning et al. developed three different algorithms: 13-layer CNN for CT slice-based predictions, a 7-layer DNNs for predictions based on clinical features, and finally, the integration of predictions from CT slices and clinical features was performed through the PLR algorithm, a regression model that evaluates one score for predicting mortality outcome [21]. Neural networks, randomization and parameter optimization, on the training dataset were performed ten times, and the model with the highest accuracy was taken into account for the final model. Moreover, to avoid overfitting, the authors opted for the dropout method, which randomly “drop out” neurons from the neural network during training. ReLU activation functions were set for both architectures, to activate the outcome of a neuron. For each method, a 10-fold cross-validation was executed ten times.

2/24 paper [36,40] used Multilayer perceptron (MLP) technique. In literature, MLP is considered a powerful machine-learning tool for medical prediction purposes, such as survival [49]. Although datasets with different origins were employed, in the study of Vaid et al. each MLP model was built with the same architecture. The MLP architecture consisted of an input layer, three hidden layers (40,10,2 units, respectively) and an output layer. In this article, the authors tried to solve the problem of data governance and privacy by training the algorithms collaboratively without exchanging the data itself, a technique known as Federated Learning (FL). The federated model was able to read the model parameters instead of raw data, thus fulfilling privacy requirements.

#### 3.5.4. Ensemble

5/24 studies implemented the XGBoost algorithm, one of the most popular ensemble method for binary classification in ML ML [22,24,35,38,39]. This classifier relies on a recursive tree-based decision system, accommodating nonlinearity and interactions between predictors, with high performance on data [24]. In Bertsimas et al., XGBoost was chosen thanks to its capability of reducing the system complexity [22]. Vaid et al. decided to use the same algorithm with a first dataset containing missing subjects’ data values, and a second dataset, in which features with >30% missing values were dropped and k-nearest neighbors were used to input missing data in the remaining feature space space [24]. Yan et al. carried out a different features selection, obtaining the final six significant features and used them for the training of the model defined as “simple-tree XGBoost” [38]. A total of 3/24 studies chose the Gradient Boosting Decision Tree (GBDT) algorithm algorithm [30,42,43]. The biggest difference with XGBoost is that the latter uses a more regularized model objective function to prevent overfitting. The studies compared this algorithm with other non-ensemble learners, including LR, SVM and neural networks. Yu et al. implemented a new gradient-boosting algorithm, CatBoost (https://catboost.ai/, 31 May 2021) that has the ability to encode categorical features. Rozenbaum et al. tested the LightGBM classifier, a novel GBDT algorithm able to accelerate the training process [41,50]. A total of 7/24 works decided to test Random Forest (RF) ensemble algorithm algorithm [20,23,28,34,37,40,42]. RF is another ensemble learning model characterized by multiple decision trees and considered as one of the best-performing learning algorithms [28]. An et al. and used RF to select the predictors before the final training [34,37]. Gao et al. developed an ensemble model based on the best performance obtained from baseline ML models, including LR, SVM, KNN, GBDT, and NN, on an internal validation cohort with 10-fold cross-validation to tune model parameters [33]. To improve the model’s ability to recognize minority categories, they raised the weights of the minority class category in the model, increasing the punishment for the wrong classification of minority categories during training. Once the best predictive performance was achieved, an ensemble model derived from four baseline models (LR, SVM, GBDT, and NN), was proposed for prediction by assigning weights manually on each individual estimator. In order to improve the mortality prediction, Ko et al. created a new ensemble AI model combining a 5-layer DNN and an RF model, named EDRnet (ensemble learning model based on DNN and RF models) [27]. The structure included a DNN architecture with one input layer and 30 features, including 28 biomarkers, age and gender. The input layer was fed into three consecutive dense layers consisting of 30, 16 and 8 neurons, respectively. To avoid overfitting, the authors applied the dropout method. Finally, the last fully connected layer was fed into a softmax layer, providing probabilities for patient mortality as output. Separately, the authors trained an RF model using a maximum feature number of five. Soft voting was implemented to obtain the final predicted mortality probability value, starting from DNN and RF results. In particular, soft voting consists of the average of the two probability values p(DNN) and p(RF), if the value is greater than or equal to 0.5, then the prediction result represents death; otherwise, it represents survival. As already mentioned, Subudhi et al. are the only ones to test a very high number of baseline ML algorithms (18) including the known ensemble learners GBDT, XGB, RF, and others such as: AdaBoost classifier, Bagging classifier, Extra trees classifier and Gaussian process classifier.

### 3.6. Metrics

None of the articles chose to evaluate ACC, AUC-ROC, AU-PRC, SENS, SPEC, PPV, NPV, MCC, and B-ACC altogether. The 10-fold was the most frequent cross-validation method [21,24,25,26,28,33,34,37,39]. In Table 3 we report the high-performing ML techniques and corresponding metrics’ values for each paper, detailing whether the result is referred to as a kind of validation. For those works lacking a performance report, we did not show any values in the table.

## 4. Discussion

Few studies attempted COVID survival analysis with statistical methods [34,51,52,53,54,55]. We decided to focus our review on mortality prediction through ML techniques which are able to fit nonlinear and complex interaction effects between predictors [56]. Particularly, ML improved predictability compared to other statistical methods on prediction of survival, in various practical domains [56,57]. Variability in dataset dimensions, experimental methods and features choices limit the comparison of the selected studies.

### 4.1. Datasets

The studies included in this review share several limitations. First, the number of patients available for testing might be considered small, affecting the significance of the results. Additionally, deceased cases are often a minority compared to the ones alive. The few datasets that are publicly available are subject to the possible risk of institutional bias [13] due to the lack of information about exclusion criteria. An additional bias could be related to the impossibility of knowing whether patients are truly SARS-CoV-2 positive due to the unclear definition of patients recruitment [13]. In addition, most studies were blind to patients who were admitted for clinically suspected SARS-CoV-2 and tested positive for the virus but died due to unrelated morbidities. Since imbalance issues characterize the SARS-CoV-2 mortality rate 3.6% (https://coronavirus.jhu.edu/data/mortality, 31 May 2021) (Table 1), unbalanced data selection may positively or negatively affect the performance of the training and testing process [24,26,28,43]. It is known from the literature that a representative sample is required for a stable model [58]. Nevertheless, these good results may be due to the adopted methods (Neural networks, SVM, Ensemble algorithms) that are known from previous literature to achieve high performance on unbalanced datasets adjusted with oversampling or undersampling techniques [59,60,61,62]. Subudhi et al. adopted a random undersampling, comparing the excluded patients of the majority class with patients included to ensures that none of the features were significantly different (*p* ≥ 0.05) [40].

### 4.2. Demography

Although the implemented models are representative of hospitalized patients with confirmed SARS-CoV-2 infection and relative outcome within the geographic remit of the study site, caution should be used when generalizing to other populations. Particularly, results may not be generalized to populations with different geographical and socioeconomic conditions, differences in national health service or insurance-based health expenses. A merged database and a prospective validation could be useful in a target population generalization. Furthermore, caution should be exercised in management practices changes or evolution of COVID pathogenesis [40].

### 4.3. Accuracy and AUC-ROC

Looking at the performance measures of the developed models, only a few achieved ACC > 90% on at least one validation technique [24,27,33,35,43]. The highest accuracy for internal (99.1%) and external (99.7%) validation was achieved by Guan et al. with XGBoost. In terms of ML methods, ensemble learning was high performing (ACC > 90%) among studies(Gao et al. 2020; Ko et al. 2020; Vaid et al. 2020; Guan et al. 2021; Stachel et al. 2021). Moreover, studies that compared non-ensemble and ensemble learners showed best performance with ensemble models [22,24,27,30,33,35,39,40,42,43]. This is in line with ensemble learning being recognized as superior in terms of prediction performance to individual models [57]. Moreover, ensemble models are less prone to overfitting issues compared to individual classifiers [63,64]. A total of 3/24 studies reported ACC > 80% and AUC-ROC > 80%, but no information on K-fold cross-validation was available. K-fold cross-validation is important to achieve higher accurate results with a limited amount of data [65]. Moreover, Wong et al. suggested to repeat K-fold cross-validation several times in order to obtain reliable accuracy [66]. A total of 7/24 studies reported prediction performance with internal and external validation contributing to the model training generalization on a wide target population [22,23,24,27,30,33,35,37].

### 4.4. Other Metrics

In most of the selected studies SENS and SPEC, which provide information about the ability to detect deceased or cured cases, exceeded 70% on at least one validation [25,27,29,30,34,36,37,39,42]. Only a limited number of studies (9/24) indicated predictive values (PPV and NPV) [21,22,25,26,30,32,34,39,43], despite these being considered important information for performance prediction assessment, on a par with sensitivity and specificity [67]. Considering previous observations, the use of only the most common metrics, such as AUCROC and ACC, limits the model validation. Definitely, all the metrics mentioned above are required in a machine learning study to allow a complete view of the performance of the final model, so as to assess whether the result truly represents a performing model given the size of the dataset and its imbalance.

### 4.5. Mortality Prediction within Different Times

Three studies tested model performance for death prediction within different times from admission: 3, 5, 7 hospital days in the study by Vaid et al.; 0, 10, 35 days in the study by Yan et al.; 7 days after admission and 7 days prior to discharge in Stachel et al. [24,38,43]. Vaid et al. achieved a better prediction (in terms of ACC, AUC-ROC) of mortality events within 3 days from admission, suggesting a role of the ensemble learner in the identification of patients at immediate mortality risk [24]. Additionally, Yan et al. and Stachel et al. reported that the ACC value of the prediction increases closer to the patient’s outcome, suggesting that deteriorations of patient’s conditions could give an early warning to the clinicians [38,43]. According to Ikemura et al., it would also be interesting to test models performance for predicting the death of patients within two distant timeframes (e.g., the first week of admission and the fourth week after admission) [39].

### 4.6. Models Validation

Vaid et al. and Subudhi et al. reported that prospectively merged validation performance dropped compared with the internal validation [24,40]. The interest in the development and validation of prediction models in clinical setting is growing [68,69], but 14/24 studies of our review reported prediction performance with internal validation only [20,21,25,26,28,29,30,31,32,34,36,38,39,42]. Furthermore, external validation is a rigorous key step before disseminating the prediction model in a clinical setting [70,71]. Since the aim of the reported predictive models is to inform patients and carriers about a mortality outcome, it is essential that predictions should be well-calibrated on a target population [72,73]. In this context, an external validation could contribute to extend this target population and to generalize the model. For this reason, measures about calibration (i.e., Z-statistic) should be considered [74] in addition to discriminate data into classes via metrics such as the AUCROC and ACC.

### 4.7. Clinical Features Predictive Ability

The ability to enhance prognostication through the integration of biomarkers in the clinical practice moves the medical field towards personalized medicine, as well as improving treatment strategies [75]. In this review, we identified the most significant biomarkers through features ranking techniques. Our analysis revealed that all the models were fed with binary and continuous features and all studies included laboratory parameters with a single exception [33]. Due to the retrospective nature of the studies, some implemented models do not include potentially important predictors of mortality outcome, such as comorbidities, vital signs, treatment, laboratory and radiological features. In addition, several studies have missing features for some subjects. Missing values are a challenging problem in SARS-CoV-2 baseline ML and DL model development [76,77,78]. Particularly some of the variables might be deleted during data pre-processing with the consequence of underestimating their role in predicting patients outcomes [37]. To overcome these limitations, it would be necessary to standardize relevant features in a prospective multicenter analysis. Among the features with higher ranking, there are Age, CRP, LDH (Table 2). A significant association between older age and SARS-CoV-2 infection mortality was observed in other literature without ML [79]. Moreover, the serum LDH level was found to be an independent risk factor for both severity and mortality in COVID patients [80]. Rastad et al. reported the CRP level as an additional risk factor [81]. Although the studies showed some variability in the feature extraction techniques, most of them have revealed a highly significant association between the feature’s age, CRP, LDH and mortality [22,27,30,35,36,38,39,41]. Among the experiments that use ensemble methods [22,24,27,28,33,35,38,39,40,41,42,43], the ones using the features CRP, LDH and age (after features ranking) obtained the best performing results [24,27,35]. Moreover, using these features, Vaid et al. compared ensemble model and non-ensemble models (LR and LASSO), obtaining the best performance with the former. This finding highlights the predictive power of the combination between high predictive features (Age, CRP and LDH) and ensemble models. Since the sample size is often imbalanced with a relative minority of COVID positive mortalities, it might be useful to create a worldwide database for the generalization of results and the most important extracted features, with a well-balanced number of survivors and non-survivors. Finally, it is important to note that the SARS-CoV-2 pandemic is unusual and evolving. Therefore, a real-time update of model prediction capabilities would be required.

### 4.8. Images Features Predictive Ability

Only two of the studies had information regarding radiologic images. Imaging may also be an important prognostic factor. The results obtained from Ning et al. and Fang et al. in terms of accuracy (78% reported by Ning et al.) and AUC-ROC (85% and 73.6% respectively) [21,23] are worse than others that used clinical features only. This could depend on a lower number of deceased patients (8% of total subjects in Ning et al. and not specified in Fang et al.). Moreover, the mortality analysis of Ning et al. included laboratory features and imaging features only [21] and Fang et al. included only score features related to CT images. On the other hand, Ning et al. reported a good MCC value (53%) that could be related to the integration of the DL technique and images features. According to recent studies [38], chest CT images play an important role in the diagnosis, monitoring and severity stratification of COVID [44]. The results reported by Ning et al. and Fang et al. showed that images related features are not best performing [21,23]. However, further studies with a dataset of clinical features and images should be created to fully exploit the benefits of integrating clinical and imaging features. Although different studies used X-rays for predicting mortality, with both radiologist-assessed [53,54] and AI-assessed [55] disease severity scores, in our knowledge, there are no studies that applied these predictors to ML methods in the evaluation of mortality. Further studies could evaluate the usefulness of this application

## 5. Conclusions

This systematic review specifically considers the state of the art in ML and DL as applied to COVID mortality prediction. Both binary and multi-class features are considered throughout the review. We summarized the developed models considering data source, data partitioning, class of features, ML technique and evaluation metrics for performance assessment. Clinical features are used in all studies for data samples, while only one paper currently has CT images features. Most of the studies presented an imbalanced number of survived and non-survived cases. We found some best practices that studies could follow for developing optimal ML models: (1) the use of a high-quality dataset with a large balanced number of samples, (2) the implementation of an ensemble of different ML methodologies, (3) clinical features should include different features class type including Age, CRP, LDH values, (4) as many metrics as possible should be reported to have a complete view on model performance, including both the most common metrics, such as AUCROC and ACC, and other important metrics for performance prediction assessment, such as SENS, SPEC, PPV and NPV.

The considerations in this review may help to develop further studies to predict mortality in COVID patients, including both adulthood and childhood, although children and young people remain at low risk of COVID mortality [82]. Moreover, suggestions collected in this study could also be useful to predict prognoses other than mortality (e.g., intubation and length of hospital stay).

## Figures and Tables

**Table 1:** Studies Data Sources, Samples and Validation Characteristics.

Reference	Centers	Location	Survived and Non-Survived Sample Size	Type of Validation	Sample Size Train	Sample Size Test	Online Available Dataset
[24]	Five centers	United states	2392 survived, 1323 non survived (deaths up to 3 gg:140; 5 gg: 281; 7 gg: 393;10 gg: 509)	Internal, External, Internal Prospectively, Prospectively Merged	1514 (deaths up to 3 gg: 40; 5 gg: 74; 7 gg: 112; 10 gg: 182) (center1)	external: 2201 (deaths up to 3 gg:135; 5 gg:276; 7 gg: 382; 10 gg:494) (center 2, 3, 4, 5; time1) prospectively merge: 383 (deaths up to 3 gg: 3; 5 gg: 5; 7 gg: 11; 10 gg:15) (all five center merged; time2)	NO
[25]	Single center	United Kingdom	275 survived, 93 non survived	Internal	318	80	NO
[26]	Single center	United Kingdom	275 survived, 93 non survived	Internal	318	80	NO
[27]	Four centers	Korea	299 survived, 214 non survived	Internal, External	361 (195 survived, 212 non survived) (center1)	external: 106 (survived 104, non survived 2) (center2,3,4)	NO
[28]	Thirty centers	Italy	3182 survived, 712 non survived, 41.5% of whom resident in Central/Southern Italian regions (15.6% death north italy; 6.4% center-south);	Merged	2725 (all thirty centers merged)	1169 (all thirty centers merged)	NO
[29]	Single center	United states	355 survived, 43 non survived	Internal	318	80	NO
[20]	Two multicentric dataset open source	China	Cohort1: 28428 survived, 530 non survived; Cohort2: 1325 survived, 123 non survived	Two double merged	Training (1): 8687 (chort1); training (2): 434 (chort2)	double merge (1): first 14190, secondly 6081 (cohort1), double merge (2): first 710, secondly 304 (chort2)	YES
[30]	Single center	China	2737 survived, 259 non survived	Internal	2339 (center1)	internal: 585 (center1); external: 72 (70 survived, 2 non survived) (center2)	NO
[31]	Single center	China	142 survived; 39 non survived	Internal	154	27	NO
[21]	Two multicentric dataset open source	China	662 survived, 57 non survived (169933 slices)	Merged			YES
[32]	Single center	United states	2985 survived, 506 non survived	Internal	2793	698	NO
[33]	Three centers	China	1906 survived; 254 non survived	Internal, Two External	621 (535 survived, 86 non survived) (center1)	internal: 622 (533 survived, 89 non survived) (center1); external (1): 801 (741 survived, 60 non survived) (center2); external (2): 116 (97 survived, 19 non survived) (center3)	NO
[22]	Thirty three centers	Italy, Spain, United States	2302 survived; 760 non survived	Merged, Three External	2755 (25 centers merged)	merge: 760 (1:25 centers), external (1): 323 (center26), external (2): 219 (27:32 centers), external (3): 323 (center33)	NO
[34]	Multicentric database	Korea	7772 survived, 228 non survived the dataset was splitted according to the ratio 7:3	Merged	5600	2400	NO
[35]	Two centers	China	1198 survived; 72 non survived	Internal, External	554 (513 survival, 41non survival) (center1)	Internal: 233 (217 survival, 16 non survival) (center1) External: 286 (279 survival, 7 non survival) (center2)	NO
[36]	Five centers	United states	3519 survived; 510 non survived	Five Internal	Training (1): 463 (center1), training (2): 1151 (center2), training (3): 524 (center3), training (4): 378 (center4), training (5): 340 (center2)	Internal (1): 148 (center1), internal (2): 493 (center2), internal (3): 225 (center3), internal (4): 162 (center4), internal (5): 145 (center2)	NO
[37]	Two centers	China	148 survived, 99 non survived	Internal, External	183 (115 survived, 68 non survived) (center1)	external: 64 (33 survived, 31 non survived) (center2)	NO
[38]	Single center	China	(1) 298 Survivaved, 187 non survived, (2) 189 survived, 162 non survived	Internal Prospectively Internal	Training (1): 375 (time1), training (2): 246	internal prospectively (1): 110 (time2), internal (2): 105	NO
[39]	Single center	United states	3226 survived, 1087 non survived	Internal	3468	845	NO
[23]	Two centers	Iran, United States	193 patients	External	105 (center1)	88 (center1)	NO
[40]	Multicentric database	United States	5308 patients	Internal Merged Prospectively	3597 (2909 survived, 688 non survived)	1711	Researcher affiliated with Mass General Brigham may apply for access
[41]	Multicentric database	United States	648 survived, 87 non survived	Merged			NO
[42]	Single center	Italy	266 survived, 75 non survived	Internal			NO
[43]	Multicentric database	United States	2619 survived, 776 non survived	Merged Merged Prospectively	2054 (1602 survived, 452 non survived)	Internal: 477 (361 survived 116 non survived) External: 864 (656 survived 208 non survived)	NO

**Table 2:** Studies Features and Feature Ranking Techniques. Abbreviations: CRP, C-Reactive Protein; LDH, Lactate Dehydrogenase; OS, Oxygen Saturation; BUN, Blood Urea Nitrogen; RDW, Red Cell Distribution Width; DBP, Diastolic Blood Pressure; RP, Respiratory pathology; CKD, Chronic kidney disease; IHD, Ischemic heart disease; CE, Cerebrovascular event; EGFR, Estimated glomerular filtration rate; MPV, Mean platelet volume; PLCR, Platelet large-cell ratio; PT, Prothrombin time; PDW, Platelet distribution width; AA, Aspartate aminotransferase; ISR, International standard ratio; BMI, Body mass index; LOS, Lenght of stay in hospital; sBP, sistolic blood pressure; dBP, diastolic blood pressure; tAC, anticoagulation treatment; RR, Respiratory rate; MCV, Mean Corpuscolar Volume; IL-10: Interleukina—10. Yes if Included the study; No if Not Included in the study.

Reference	Features Type			Features Class						Features Selection			Dimension Reduction	Most Important Features (in Order)
	Binaries	Continuous	Images	Demographics	Commorbities	Syntoms	Vital Signs	Laboratory	Tratment	SHAP	LASSO	Others		
[24]	Yes	Yes	No	Yes	Yes	No	Yes	Yes	No	Yes	Yes	No	73 to 10	Age, Anion Gap, CRP, LDH, OS, BUN, Ferritin, RDW, DBP, Lactate
[25,26]	Yes	No	No	Yes	Yes	Yes	No	No	Yes	Yes	No	No	22	Altered mental status, Dyspnea, Age, Gender, Cough, RP, Hyperthension, Fever, CKD, IHD, CE, Myalgia, Smoking history, Cardiac failure, Days of symptoms, Obesity, Diarrea or vomiting, Anosmia e/o ageusia, Liver cirrosis, Diabetes, Abdominal pain
[27]	Yes	Yes	No	Yes	Yes	Yes	Yes	Yes	Yes	No	No	Yes	73 to 30	Lymphocytes count, Neutrophils, Albumin, LDH, Neutrophil count, CRP, Prothrombin activity, Calcium, Urea, EGFR, Monocytes, Globulin, Eosinophils, Glucose, RDW, HCO3-, RDW STD, Platelet count, MPV, PLCR, PT, Total protein, PDW, AA, Thrombocytopenia, Eosinophil count, Alkaline phosphatase, ISR, Age, Gender
[28]	Yes	No	No	Yes	Yes	No	No	Yes	No	No	No	Yes	12	EGFR, CRP, Age, Diabetes, Gender, Hyperthension, Smoking, Lung Disease, Myocardial infarction, Obesity, Hearth failure, Cancer
[29]	No	Yes	No	No	No	No	No	Yes	No	Yes	No	Yes	26 to 5	CRP, BUN, serum calcium, serum albumin, lactic acid
[20]	Yes	No	No	Yes	Yes	Yes	No	No	Yes	No	No	No	No	No
[30]	Yes	Yes	No	Yes	Yes	Yes	Yes	Yes	Yes	No	No	Yes	1224 to 83	LDH, BUN, Lymphocyte count, age, SPO2, Platlets, CRP, IL-10, HDL-C, SAO2
[31]	Yes	Yes	No	Yes	Yes	Yes	Yes	Yes	Yes	No	No	Yes	56 to 5	D-dimer, O2 index, Lymphocyte count, CRP, Doarrhea
[21]	Yes	Yes	Yes	No	No	No	No	Yes	No	No	No	No	No	No
[32]	Yes	Yes	No	Yes	Yes	No	Yes	No	Yes	Yes	No	No	34 to 20	Vented, Respiration, BMI, LOS, Race, Pulse, ICU_adm, sBP, dBP, Temp, Pressors, tAC duration in days, Steroid Treatment duration in days, Hearth, Diabetes, Cancer, Steroid, tAC, Hypertension, Gender
[33]	Yes	Yes	No	Yes	Yes	No	Yes	No	No	No	Yes	No	34 to 8	Consciousness, male sex, sputum, BUN, RR, D-dimer, number of comorbidities, age
[22]	Yes	Yes	No	Yes	Yes	No	Yes	Yes	No	Yes	No	No	22	Age, BUN, CRP, OS, Blood creatinine, Blood glucose, AA, Platelets, MCV, White Blood Cells
[34]	Yes	No	No	Yes	Yes	Yes	No	No	No	No	Yes	Yes	Not specified	Cox analysis: age > 70, male sex, disability, symptoms, infection at home; LASSO: age > 80, taking of acarbose, age > 70, taking of metformin, underlying cancer; RF: cluster infection, infection from personal contact or visit, underlying hypertension, age > 80
[35]	Yes	Yes	No	Yes	Yes	Yes	No	Yes	Yes	No	Yes	No	48 to 6	Severity, CRP, Age, LDH, Serum ferritin, IL-10
[36]	Yes	No	No	Yes	Yes	No	No	No	No	No	No	No	No	No
[37]	Yes	Yes	No	Yes	Yes	Yes	Yes	Yes	No	No	No	Yes	20 to 4	Age, CRP, Lymphocyte count and d-dimer
[38]	Yes	Yes	No	Yes	No	Yes	No	Yes	No	No	No	Yes	75 to 3	LDH, Lymphocyte count, CRP
[39]	No	Yes	No	Yes	Yes	No	No	No	Yes	Yes	No	No	48 to 10	sBP, dBP, Age, LDH, SPO2, RR, BUN, Troponin level, D-dimer level, Charlson comorbidity score
[23]	No	No	Yes	No	No	No	No	No	No	No	No	No	No	No
[40]	Yes	Yes	No	Yes	Yes	No	Yes	Yes	Yes	Yes	No	No	Not specified	EGFR, use of ventilation, Lymphocyte count, Neutrophil count, RR, procalcitonin, serum anion gap, serum potassium
[41]	Yes	Yes	No	Yes	Yes	No	No	Yes	Yes	Yes	No	No	109 to 10	Age, LDH, Ferritin, Neutrophil count, INR, Procalcitonin, CRP, Hemoglobin, AA, D-dimer
[42]	Yes	Yes	No	Yes	Yes	No	Yes	Yes	Yes	No	No	Yes	Not specified	Age, Creatinine, AA, OS, Lymphocytes, Platelets, Hemoglobin, Quick SOFA 2
[43]	Yes	Yes	No	Yes	No	No	Yes	Yes	No	No	No	Yes	142	Pulse oximetry, RR, sBP, BUN, white blood cell, Age, Length of stay, lymphocyte per cent

**Table 3:** Best performing methods and metrics results. Abbreviations: XGBoost, Extreme Gradient Boosting; ANN, Artificial Neural Network; DNN, Dense Neural Network; RF, Random Forest; SVM, Support Vector Machine; GBDT, Gradient Boosting Decision Tree; PLS, partial least square; LR, Logistic Regressor; NN, Neural Network; MLP, Multi-Layer Perceptron; LightGBM, Light Gradient Boosting Machine. No Not Included in the study.

Ref.	Machine Learning Technique	Metrics										k-Fold
		Accuracy	AUC-ROC	AU-PRC	Sensitivity	Specificity	PPV	NPV	F1-Score	MCC	Balanced Accuracy	
[24]	Ensemble (XGBoost)	int val (3 days): 97.6%; ext val (3 days): 93.6%; int val prosp (3 days): 97.1% merged val prosp (3 days): 94.2%	int val (3 days): 89%, ext val (3 days): 87.7%; int val prosp (3 days): 96.2%; merged val prosp (3 days): 87.9%	int val (3 days): 44.5%, ext val (3 days): 44.4% int val prosp (3 days): 55.1%; merged val prosp (3 days): 13.1%	int val (3 days): 44.2%, ext val (3 days): 37% int val prosp (3 days): 50% merged val prosp (3 days): 33.3%	int val (3 days):99.1%, ext val (3 days): 93.6%, int val prosp (3 days): 97.1%; merged val prosp (3 days): 94.2%	No	No	int val (3 days): 49.8%; ext val (3 days): 41.7% int val prosp (3 days): 28.6%; merged val prosp (3 days): 14.3%	No	No	10
[25]	ANN	int val 86.25%	int val: 90.12%	No	int val: 87.5%	int val: 85.9%	int val: 60.87%	int val: 96.49%	No	No	No	10
[26]	Cox Regressor	int val: 83.75%	int val: 86.9%	No	int val: 50%	int val: 96.6%	int val: 84.6%	int val: 83.6%	No	No	No	10
[27]	Ensemble (DNN + RF)	int val: 93% ext val: 92%	No	No	int val: 92% ext val: 100%	int val: 93%, ext val: 100%	No	No	No	No	int val: 93%, ext val: 96%	100
[28]	Ensemble (RF)	merged val: 83.4%	No	No	merged val: 95.2%	merged val: 30.8%	No	No	merged val: 90.4%	No	No	10
[29]	SVM	No	int val: 93%	int val: 76%	int val: 91%	int val: 91%	No	No	No	No	No	Unclear
[20]	Auto encoder	No	No	No	No	No	No	No	No	No	No	Unclear
[30]	Ensemble (GBDT)	int val (severe): 88.9%, int val (non-severe): 92.4%, int val(total): 79.9%	int val (severe): 94.1%, int val (non-severe): 93.2%, int val(total):91.8%	No	int val (severe): 89.9%, int val (non-severe): 94% int val(total):77.4%	int val (severe): 78.8%, int val (non-severe): 61.9%; int val(total):90.3%	int val (severe): 43.2% int val (non-severe):35.1% int val(total):48.3%	int val (severe):97.8% int val (non-severe): 97.9% int val(total):97.2%	No	No	No	5
[31]	DNN	No	int val: 96.8%	No	No	No	No	No	No	No	No	5
[21]	PLS	merged val: 78.73%	merged val: 85.6%	No	merged val: 88.24%	merged val: 78.26%	merged val: 16.67%	merged val: 99.26%	No	merged val: 52.36%	No	10
[32]	Ensemble (CatBoost)	int val: 80.3%	int val: 85%	No	No	No	int val: 79%	int val: 81.6%	No	No	No	Unclear
[33]	Ensemble (LR,SVM,GBDT,NN)	int val: 96.21% ext val 1: 97.6% ext val 2: 92.46%	int val:92.4%, ext val 1: 95.5%, ext val 2: 87.9%	No	No	No	No	No	No	No	No	10
[22]	Ensemble (XGBoost)	merged val: 85.02%, ext val 1: 74.92%, ext val 2:86.76%, ext val 3: 61.3%	merged val:90.19%, ext val 1:87.45%, ext val 2:91.62%, ext val 3:80.66%	No	No	merged val: 86.58%, ext val 1: 74.23%, ext val 2: 87.43%, ext val 3: 58.12%	merged val:66.3%, ext val 1:25.74%, ext val 2:48.94%, ext val 3:24.18%	merged val:93.02%, ext val 1: 97,3% ext val 2:97,09%, ext val 3:94,71%	No	No	No	Unclear
[34]	SVM	No	merged val: 96.2%	No	merged val: 92.0%	merged val: 91.8%	merged val: 25.7%	merged val: 99.7%	No	No	merged val: 91.9%	10
[35]	Ensemble (XGBoost)	int val: 99.1% ext val: 99.7%	No	No	int val: 87.5% ext val: 85.7%	No	int val: 99.1% ext val: 99.7%	No	No	No	No	500
[36]	Federate learning (MLP)	int val: 78%	int val: 83.6%	int val: 27.6%	int val: 80.5%	int val: 70.2%	No	No	int val: 32.8%	No	No	490
[37]	LR	No	int val: 89.5%, ext val: 88.1%	No	int val: 89.2% ext val: 83.9%	int val: 68.7% ext val: 79.%	No	No	No	No	No	10
[38]	Ensemble (XGBoost)	No	No	No	int val: 95%ext val prosp: 98%	No	int val: 95%ext val prosp: 91%	No	int val: 95%ext val prosp: 94%	No	No	500
[39]	Ensemble XGBoost	No	int val: 90.3%	int val: 79.1%	int val: 83.8%	int val: 83.6%	int val: 60,9%	int val: 94,4%	No	No	No	10
[23]	LR	No	ext val: 73.6%	No	No	No	No	No	No	No	No	Unclear
[40]	Ensemble (RF)	No	No	No	No	No	No	No	Int val: 87%	No	No	Unclear
[41]	LightGBM	No	merged val: 88%	No	No	No	No	No	No	No	No	10
[42]	Ensemble (RF)	No	int val: 84%	No	int val: 78.8%	int val: 77.4%	No	No	No	No	No	Unclear
[43]	Ensemble GBDT	merged val prosp: 96%	merged val prosp: 99%	No	merged val prosp: 24%	merged val prosp: 97%	merged val prosp: 90%	merged val prosp: 98%	No	No	No	Unclear

## Data Availability

Not applicable.

## Supplementary Materials

The following are available online at https://www.mdpi.com/article/10.3390/jpm11090893/s1, Table S1. Public available datasets.
